# Bio-inspired mineralization of nanostructured TiO_2_ on PET and FTO films with high surface area and high photocatalytic activity

**DOI:** 10.1038/s41598-020-70525-w

**Published:** 2020-08-11

**Authors:** Yoshitake Masuda

**Affiliations:** National Institute of Advanced Industrial Science and Technology (AIST), 2266-98 Anagahora, Shimoshidami, Moriyama-ku, Nagoya, 463-8560 Japan

**Keywords:** Chemistry, Materials chemistry, Surface chemistry

## Abstract

Nanostructured TiO_2_ coatings were successfully formed on polyethylene terephthalate (PET) films and fluorine-doped tin oxide (FTO) films in aqueous solutions. They contained an assembly of nanoneedles that grow perpendicular to the films. The surface area of the coatings on PET films reached around 284 times that of a bare PET film. Micro-, nano-, or subnanosized surface roughness and inside pores contributed to the high nitrogen adsorption. The coatings on FTO films showed an acetaldehyde removal rate of 2.80 μmol/h; this value is similar to those of commercial products certified by the Photocatalysis Industry Association of Japan. The rate increased greatly to 10.16 μmol/h upon annealing in air at 500 °C for 4 h; this value exceeded those of commercial products. Further, the coatings showed a NO_x_ removal rate of 1.04 μmol/h; this value is similar to those of commercial products. The rate decreased to 0.42 μmol/h upon annealing. NO_x_ removal was affected by the photocatalyst’s surface area rather than its crystallinity.

## Introduction

Metal oxides are useful for industrial applications such as gas sensors, batteries, and artificial photosynthesis^1–12^. For example, SnO_2_ micropatterns were fabricated on a polymer film^1^ and a silicon substrate^2^ using a patterned octadecyltrimethoxysilane self-assembled monolayer template. The sensitivity of a hydrogen gas sensor with an SnO_2_ micropattern increased linearly with increasing SnO_2_ crystallinity. SnO_2_ could also be used to fabricate a lightweight flexible gas sensor on a polymer film. A porous iron vanadate (FeVO_4_) thick film consisting of disordered nanorods was fabricated by a hydrothermal method for gas penetration and permeation^12^. The probed I–V behavior and ultraviolet–visible spectroscopy measurements confirmed the semiconducting nature of triclinic FeVO_4_ (E_g_ = 2.72 eV) and revealed that the activation energy for electric conduction was 0.46 eV. The best gas sensitivity of 0.29 ± 0.01 (m =  − 3.4 ± 0.1) was obtained at optimal working temperature of 250 °C. SnO_2_ nanowires were fabricated for an H_2_S gas sensor^3^. They were functionalized with copper particles during chemical vapor deposition. A CuO@SnO_2_ p–n heterojunction was fabricated by oxidizing Cu to CuO. The fabricated sensor demonstrated high sensitivity and selectivity for H_2_S gas. VO_2_ nanobelts or nanoparticles were fabricated by a hydrothermal method for thermochromic devices^4^. Nanoparticles showed high phase transition enthalpy (ΔH = 32.4 J/g) and VO_2_-PET composite films showed optical switching characteristics (T_lum_ = 33.5%, ΔT_sol_ = 16.0%). A dye-sensitized solar cell was prepared with a ZnO@TiO_2_ core shell nanorod array via a low-temperature solution method^5^. High-aspect-ratio ZnO nanorod arrays were covered with a TiO_2_ shell. The TiO_2_ shell increased the short circuit current (from 4.2 to 5.2 mA/cm^2^), open circuit voltage (from 0.6 to 0.8 V), fill factor (from 42.8 to 73.02%), and overall cell efficiency (from 1.1 to 3.03%). ZnO nanorods were grown on a highly conductive sandwich-like seed layer (ZnO seed layer/Ag nanowires/ZnO seed layer) and modified with α-Fe_2_O_3_ nanoparticles^6^. ZnO nanorods showed high potential for Ca^2+^ sensing in water or serum samples. They can be applied to drinking and irrigation water as well as to clinical analysis. A sensitive and selective sunlight-driven photoelectrochemical sensor was developed for the direct detection and reduction of chromium(VI)^11^. It was based on single-crystal rutile TiO_2_ nanorods decorated with gold nanoparticles. It showed high sensitivity under solar simulator illumination. Evaluations of real water samples showed excellent anti-interference and recovery capabilities. Metal oxide nanomaterials and a biosensor fabricated using them were reviewed^7^. Nanomaterial deposition on conductive electrodes is a crucial step for achieving high performance, and a simple, stable, and reproducible method is considered essential for depositing nanomaterials for fabricating a biosensor.

Many devices have reaction sites on the metal oxide surface, and therefore, they need to have a large surface area. In addition, studies are developing new devices by using the characteristic surface structure of metal oxides. Controlling the size, shape, and crystallinity of the metal oxide is known to greatly affect the device characteristics, and new metal oxide materials are being actively developed. There is also a strong need to control the shape and even the exposed crystal plane after the metal oxide material is crystallized. Especially, nanostructured TiO_2_ films with high surface area and high photocatalytic activity are strongly required for photocatalysts and related devices. Performance of the nanostructured TiO_2_ films is strongly affected by the size, shape, crystallinity, and the exposed crystal plane. The development of high performance nanostructured TiO_2_ films is a powerful demonstration of precise control of the size, shape, crystallinity, and the exposed crystal plane.

Metal oxide nano-/microstructures have also been synthesized in animals and plants. The size, shape, crystallinity, and surface structure of metal oxides in aqueous solutions were controlled at room temperature and atmospheric pressure. They are called “Bio-mineralization” and have created a new academic field, “Bio-inspired mineralization”. In the bio-inspired mineralization, we learn from nature and aim to develop novel materials that are not in nature.

This study investigates the bio-inspired mineralization of nanostructured TiO_2_. Nanostructured TiO_2_ was formed on polymer or inorganic films in aqueous solutions. Further, its surface area and photocatalytic properties were investigated.

## Experimental

Ammonium hexafluorotitanate ([NH_4_]_2_TiF_6_) (Morita Chemical Industries Co., Ltd., FW: 15, purity 96.0%) and boric acid (H_3_BO_3_) (Kishida Chemical Co., Ltd., FW: 61, purity 99.5%) were used as received. Nanostructured TiO_2_ films were crystallized on polyethylene terephthalate (PET) films or fluorine-doped tin oxide (FTO) films in an aqueous solution containing ammonium hexafluorotitanate (50 mM) and boric acid (150 mM) at 50 °C for 24 h.

The morphology of the nanostructured TiO_2_ film on the PET film was observed using a field-emission scanning electron microscope (FE-SEM; JSM-6335F, JEOL Ltd.) at 20 kV. Nitrogen adsorption–desorption isotherms were obtained using Autosorb-1 (Quantachrome Instruments). Nanostructured TiO_2_ films on PET films were outgassed at 110 °C under 10^–2^ mmHg for more than 6 h prior to measurement. The specific surface area was calculated by the Brunauer–Emmett–Teller (BET) method using adsorption isotherms. The pore size distribution was calculated by the Barrett–Joyner–Halenda (BJH) method using desorption isotherms. The photocatalytic properties of the nanostructured TiO_2_ on FTO films were evaluated at the Kanagawa Academy of Science and Technology (KAST), Japan, based on Japanese Industrial Standards (JIS).

## Results and discussion

### *Bio-inspired mineralization of nanostructured TiO*_*2*_

Ten sheets of PET films (50 mm long × 50 mm wide × 0.1 mm thick) were pasted on glass plates with polytetrafluoroethylene tapes. Ammonium hexafluorotitanate (10.31 g) and boric acid (9.321 g) were dissolved in 1,000 mL of distilled hot water^13^. The concentrations of ammonium hexafluorotitanate and boric acid were 50 and 150 mM, respectively. PET films were exposed to vacuum-ultraviolet light for 20 min in air using a low-pressure mercury lamp (PL16-110, SEN Lights Co.). They were immersed in the solutions at 50 °C for 24 h. The titanium oxide-formed surface of the PET films faced obliquely downward. Therefore, even if homogeneously nucleated titanium oxide particles formed in the solution settle, they are less likely to be deposited on the PET film. Further, the PET film is less likely to be bent or deformed in the solution because it is attached to the glass plate. Moreover, titanium oxide is formed only on the PET film surface because the back surface of the film is in close contact with the glass plate. Thereafter, the glass plates were removed from the solution. The PET films were peeled off from the glass plates, washed with running water, and dried under a strong air flow.

### *Nanostructured TiO*_*2*_* with high surface area*

**Figure 1:**
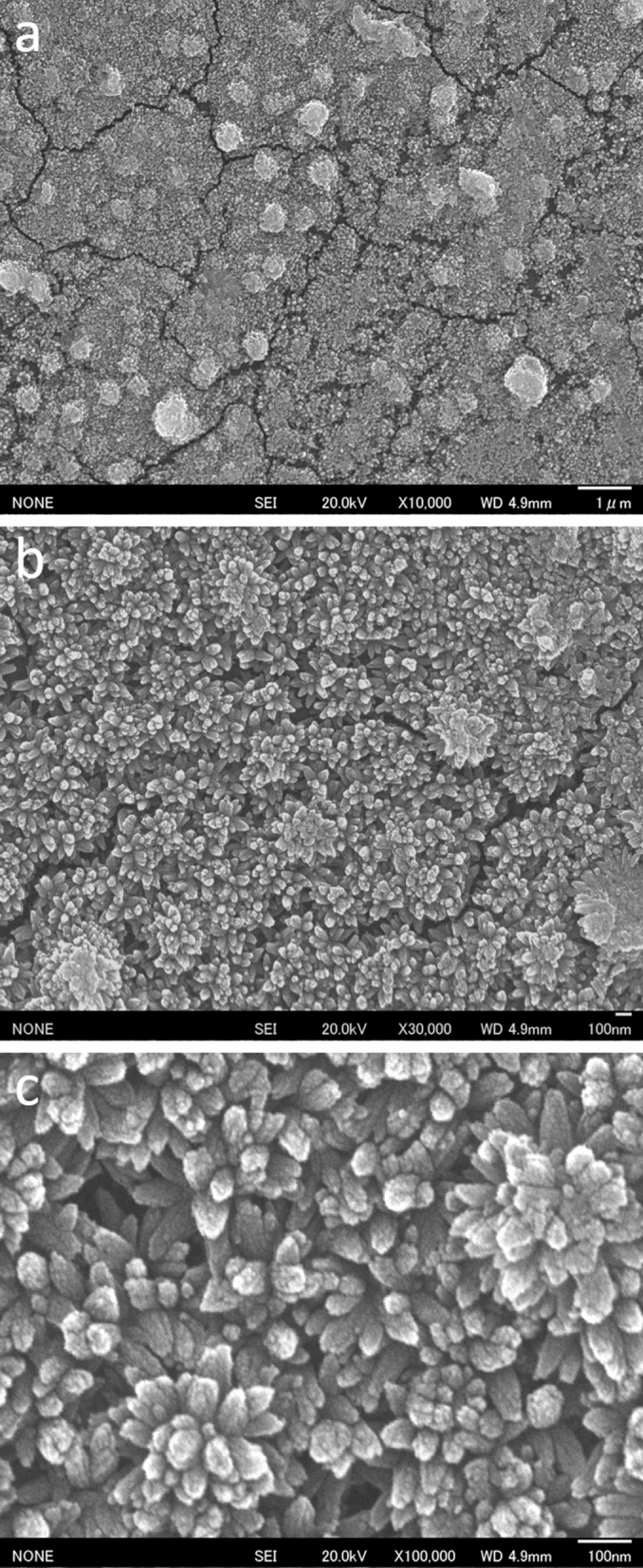
(**a**) FE-SEM micrograph of nanostructured TiO_2_ on a PET film. The nanostructured TiO_2_ was formed in an aqueous solution using liquid-phase crystal growth. (**b**), (**c**) Magnified FE-SEM micrographs of (**a**).

PET films were uniformly covered with nanostructured TiO_2_ (Fig. 1a). The coatings had an uneven surface structure (Fig. 1b). The needle-like surface structures had size of ~ 5–10 nm (Fig. 1c). Each needle had nanosized surface asperities.

The mass of the PET film was measured before and after immersion in the aqueous solution. The mass of nanostructured TiO_2_ was calculated from the difference in mass before and after immersion. The composite was cut to obtain rectangular pieces with dimensions of ~ 3 mm × 10 mm. All of these pieces were filled in a glass sample holder for gas adsorption measurements. The gas adsorption amount of the composite can be measured with almost no influence of the PET film, and the measured gas adsorption amount of the composite can be regarded as the gas adsorption amount of nanostructured TiO_2_.

**Figure 2:**
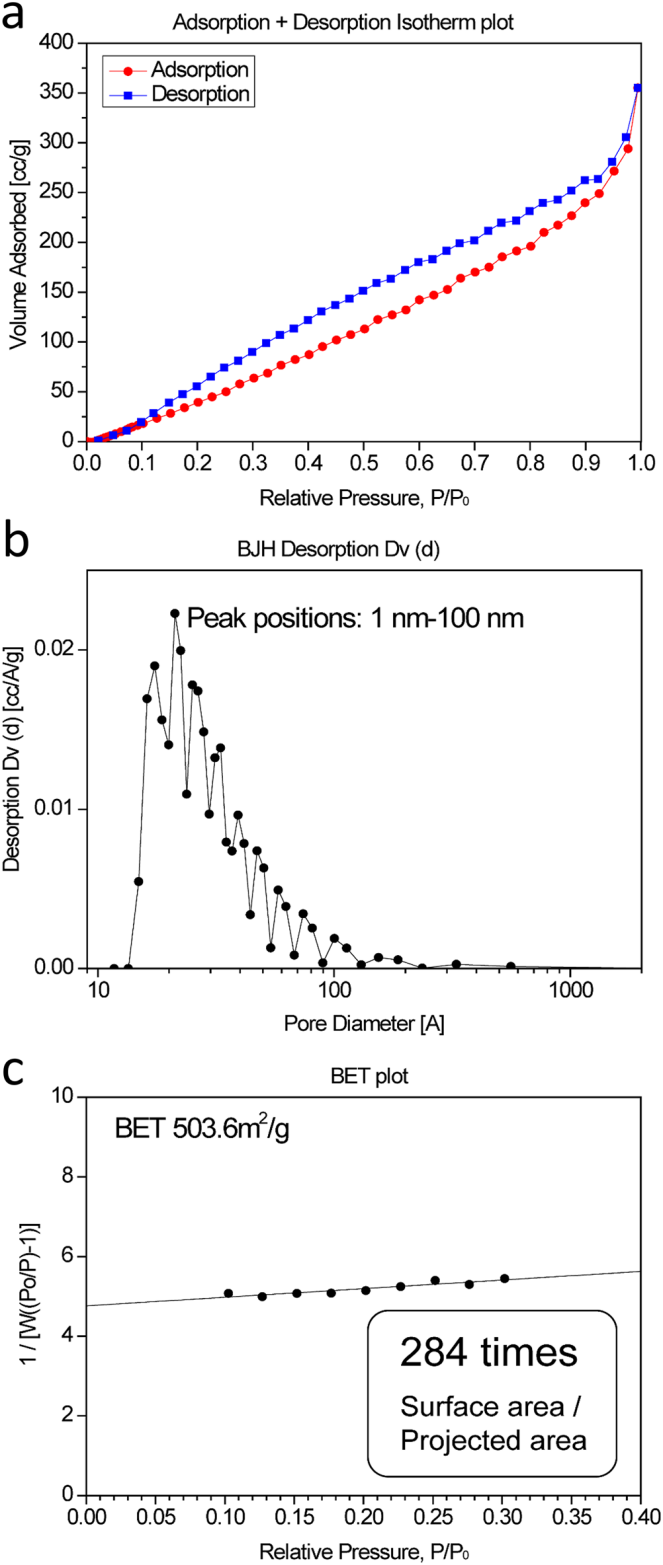
(**a**) N_2_ adsorption–desorption isotherm of nanostructured TiO_2_ on a PET film. (**b**) Pore size distribution calculated from N_2_ adsorption data of nanostructured TiO_2_ on a PET film using BJH equation. (**c**) BET surface area of nanostructured TiO_2_ on a PET film. Inset: ratio of surface area to substrate projected area.

Nanostructured TiO_2_ showed nitrogen adsorption–desorption isotherms (Fig. 2a). The pore size distribution of nanostructured TiO_2_ was calculated from the nitrogen desorption isotherm by the BJH method (Fig. 2b). Nanostructured TiO_2_ had inside pores and/or interparticle spaces of ~ 2–100 nm. The result did not indicate whether nanostructured TiO_2_ has pores of 1 nm or less because such pores cannot be estimated by the BJH method. The BET specific surface area was calculated to be 503.6 m^2^/g (Fig. 2c). The total surface area of nanostructured TiO_2_ was calculated by multiplying this value by the mass of nanostructured TiO_2_. The ratio of the surface area to the substrate projected area was calculated to around 284 times that of a bare PET film by dividing the total surface area of nanostructured TiO_2_ by the projected area of substrates (25,000 mm^2^, 50 mm × 50 mm × 10 sheets). The total surface area is not affected by the error of the weight of the nanostructured TiO_2_ in this calculation method, and therefore, it can be determined accurately. To the best of the authors’ knowledge, the TiO_2_ film surface area was the highest ever reported. Nanostructured TiO_2_ in this study was considered to be different from general nanostructured TiO_2_ reported elsewhere. Micro-, nano-, and subnanosized surface roughness and inside pores contributed to increased nitrogen adsorption. Surface crystal defects such as kinks, terraces, and secondary nuclei were also considered to contribute to increased nitrogen adsorption.

### *Nanostructured TiO*_*2*_* with high photocatalytic activity*

FTO films were exposed to vacuum-ultraviolet light for 20 min. They were immersed in the solutions containing ammonium hexafluorotitanate (50 mM) and boric acid (150 mM) at 50 °C for 24 h. The photocatalytic properties of the nanostructured TiO_2_ on FTO films were evaluated based on JIS R 1701-2: 2008 (acetaldehyde removal performance) and JIS R 1701-1: 2010 (nitrogen oxide (NO_x_) removal performance). Acetaldehyde and NO_x_ serve as evaluation indices for indoor and outdoor photocatalytic activity, respectively. Materials with high photocatalytic activity for acetaldehyde and NO_x_ can be used in building interiors and exteriors, respectively. The photocatalytic characteristics were evaluated using two FTO films (50 mm × 26 mm × 1.1 mm in thickness) (AGC Fabritech Co., Ltd., TOC substrate (DU film)). The FTO layer (0.1 mm in thickness) was formed on a glass substrate (0.1 mm in thickness). The size of the test piece was around half of the JIS-specified size (50 mm × 100 mm). The measured values excluding regeneration efficiency for NO_x_ removal characteristics were thus doubled.

**Figure 3:**
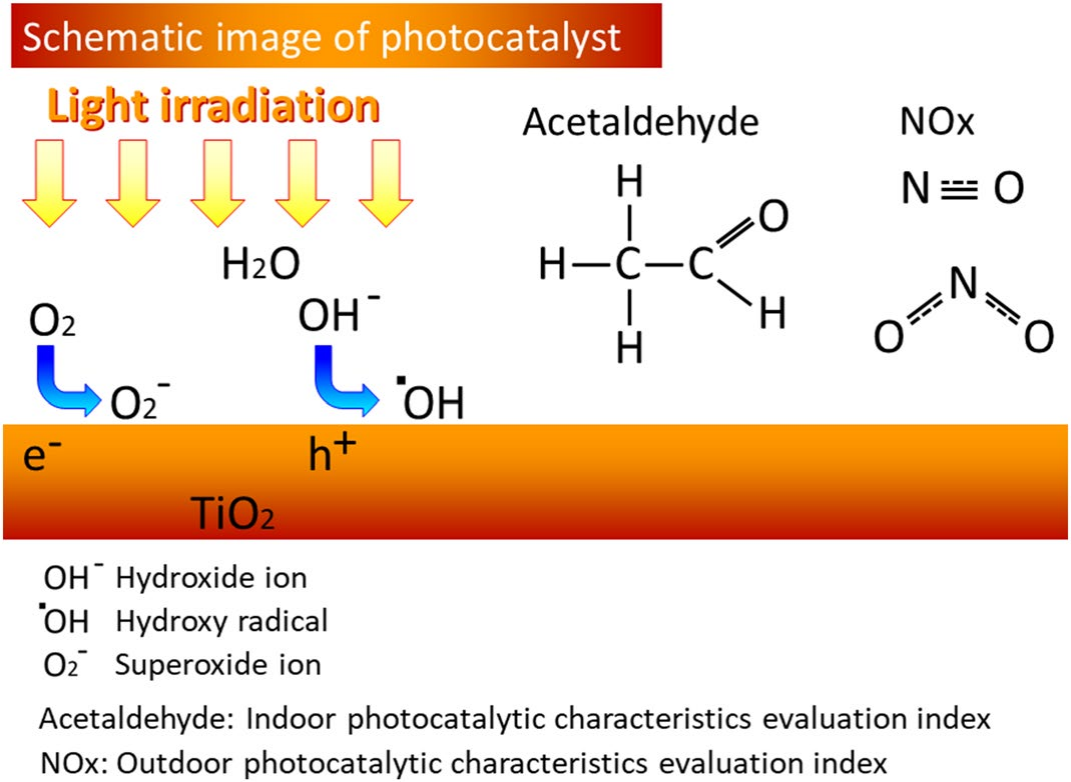
Schematic image of photocatalytic activity.

In general, light irradiation on a photocatalytic material such as TiO_2_ generates electrons and holes on the surface (Fig. 3). Oxygen and water in the air react with electrons and holes, respectively. These reactions produce hydroxy (OH) radicals and superoxide ions on the titanium dioxide surface. OH radicals have strong oxidizing power and remove electrons from acetaldehyde molecules. Acetaldehyde molecules lose electrons and break bonds. They were converted to CO_2_ and/or H_2_O that were released to the atmosphere.

**Figure 4:**
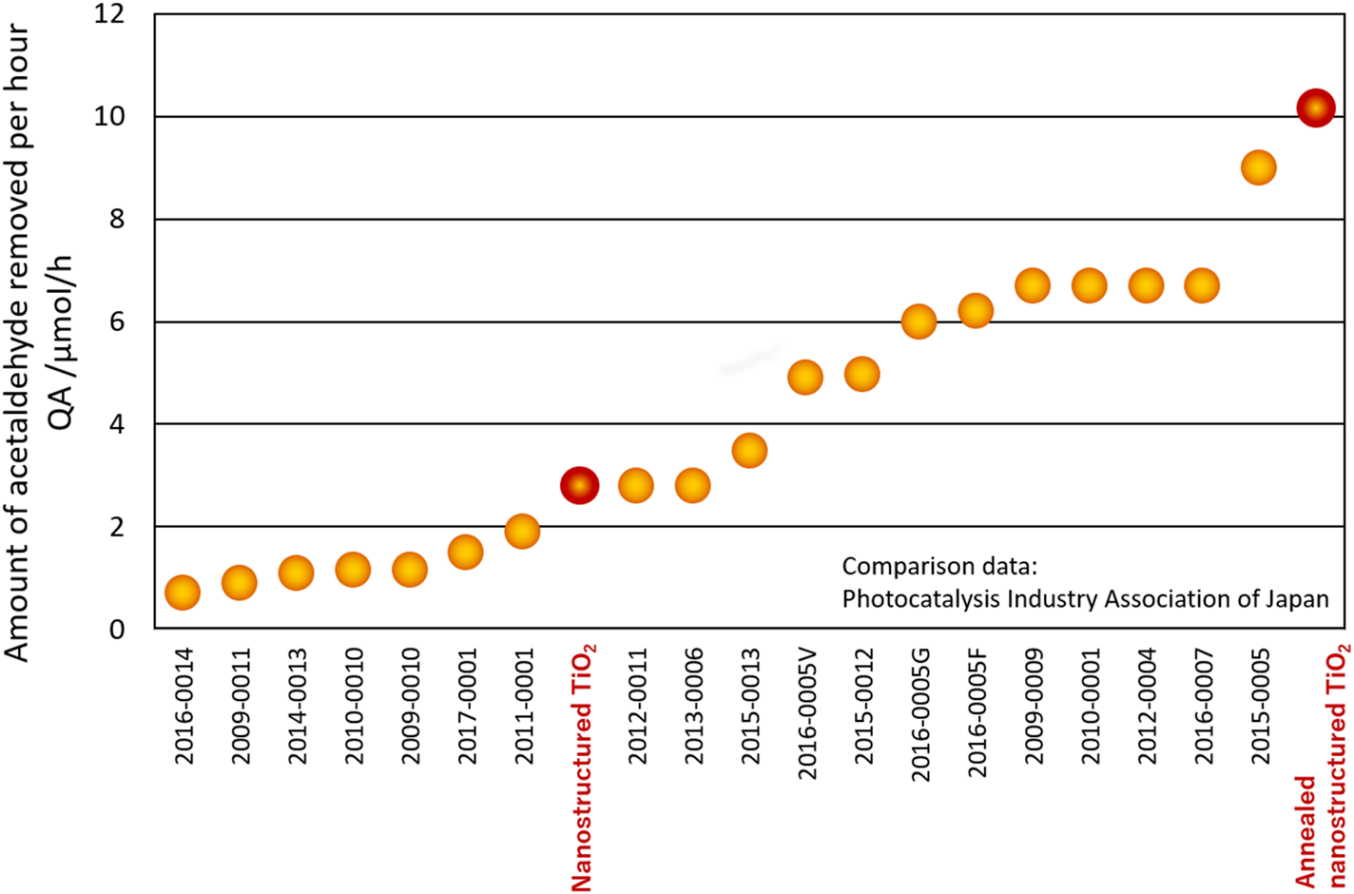
Comparison of acetaldehyde decomposition characteristics with those of commercial products certified by the Photocatalysis Industry Association of Japan.

Acetaldehyde was removed from the sample at the rate of 2.80 μmol/h, and its removal ratio was 20.6% (Table 1). This rate was similar to that of commercial products certified by the Photocatalysis Industry Association of Japan^14^ (Fig. 4). Further, acetaldehyde was converted to CO_2_ at the rate of 4.56 μmol/h, and its conversion ratio was 16.8%.

**Table 1:** Photocatalytic properties of nanostructured TiO_2_.

	Acetaldehyde decomposition characteristics				NO_x_ decomposition characteristics								
	QA/μmol/h	QC/μmol/h	RA/%	RC/%	QNO_x_	Qads	QNO	QNO_2_	Qdes	ηw	Qw1	Qw2	QNo_x_
TiO_2_	2.80	4.56	20.6	16.8	1.04	0.08	3.0	1.24	0.8	1,400	12.5	2.42	1.04
TiO_2_ (after annealing at 550 °C for 4 h)	10.16	16.84	74.8	60.2	0.42	0.08	2.7	1.68	0.7	2,100	7.34	1.28	0.42

They were evaluated based on JIS R 1701-2: 2008 (left, acetaldehyde removal performance) and JIS R 1701-1: 2010 (right, NO_x_ removal performance).

**Figure 5:**
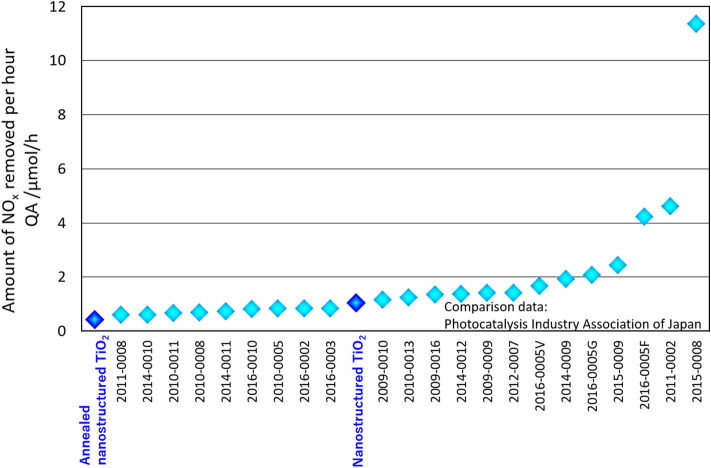
Comparison of NO_x_ decomposition characteristics with those of commercial products certified by the Photocatalysis Industry Association of Japan.

The amount of NO_x_ removed was 1.04 μmol (Table 1). This value was similar to that of commercial products (Fig. 5). The NO_x_ adsorption and desorption amounts were 0.08 and 0.8 μmol, respectively. The amount of nitric oxide removed was 3.0 μmol. The amount of nitrogen dioxide generated was 1.24 μmol.

The regeneration efficiency upon washing with water was 1,400% (no conversion). The first and second elution amounts of NO_x_ were 12.50 and 2.42 μmol, respectively. The regeneration efficiency exceeded 100%, suggesting that NO_x_ was generated from the sample during the test and was added to the NO_x_ elution amount. This result indicated that nanostructured TiO_2_ contained nitrogen inside and/or on its surface. Ammonium hexafluorotitanate is one of the sources of nitrogen.

### *Annealed nanostructured TiO*_*2*_* with high photocatalytic activity*

Nanostructured TiO_2_ on FTO films were annealed at 500 °C in air for 4 h. In general, high-temperature treatment was considered to improve crystallinity but reduces internal vacancies and surface defects. Photocatalytic properties are affected by both changes.

Notably, the acetaldehyde removal rate increased greatly from 2.80 to 10.16 μmol/h upon annealing, and the removal ratio was 74.8% (Table 1). The rate exceeded that of commercial products (Fig. 4). This was one of the advantages of nanostructured TiO_2_. Further, acetaldehyde was converted to CO_2_ at the rate of 16.84 μmol/h, and its conversion ratio was 60.2%.

The NO_x_ removal rate decreased from 1.04 to 0.42 μmol/h upon annealing (Table 1, Fig. 5). NOx removal was affected by the photocatalyst’s surface area rather than its crystallinity. The NO_x_ adsorption and desorption amounts were 0.08 and 0.7 μmol, respectively. The amount of nitric oxide removed was 2.7 μmol. The amount of NO_x_ generated was 1.68 μmol. The regeneration efficiency upon washing with water was 2,100% (no conversion). The first and second elution amounts of NO_x_ were 7.34 and 1.28 μmol, respectively.

## Conclusions

Nanostructured TiO_2_ was formed on PET films in aqueous solutions. Nanostructured TiO_2_ coatings showed high N_2_ adsorption. The BET specific surface area was ~ 503.6 m^2^/g and the ratio of the surface area to the substrate projected area reached around 284 times that of a bare PET film. Furthermore, the photocatalytic property of nanostructured TiO_2_ on FTO films was evaluated. The acetaldehyde removal rate increased greatly from 2.80 to 10.16 μmol h upon annealing. This value exceeded that of commercial products (Fig. 4). The amount of NO_x_ removed was 1.04 μmol; this was similar to that of commercial products. This value decreased to 0.42 μmol/h upon annealing. NO_x_ removal was affected by the photocatalyst’s surface area rather than its crystallinity. The study results revealed that nanostructured TiO_2_ had different nanostructure and surface conditions. These contributed to the high surface area and high photocatalytic activity. In particular, the acetaldehyde decomposition characteristics were higher than those of commercial products. Nanostructured TiO_2_ can be used for low-cost, large-area coating of various substrates such as polymer films. The high surface area and high photocatalytic activity can be used for various applications to building interior materials.
